# Wound Healing in PatientsWith Cancer

**Published:** 2008-01-11

**Authors:** Wyatt G. Payne, Deepak K. Naidu, Chad K. Wheeler, David Barkoe, Marni Mentis, R. Emerick Salas, David J. Smith, Martin C. Robson

**Affiliations:** Institute for Tissue Regeneration, Repair, and Rehabilitation, Bay Pines VA Medical Center, Bay Pines, Florida, and The Division of Plastic and Reconstructive Surgery, University of South Florida, Tampa

## Abstract

**Objective:** The treatment of patients with cancer has advanced into a complex, multimodal approach incorporating surgery, radiation, and chemotherapy. Managing wounds in this population is complicated by tumor biology, the patient's disease state, and additional comorbidities, some of which may be iatrogenic. Radiation therapy, frequently employed for local-regional control of disease following surgical resection, has quantifiable negative healing effects due to local tissue fibrosis and vascular effects. Chemotherapeutic agents, either administered alone or as combination therapy with surgery and radiation, may have detrimental effects on the rapidly dividing tissues of healing wounds. Overall nutritional status, often diminished in patients with cancer, is an important aspect to the ability of patients to heal after surgical procedures and/or treatment regimens. **Methods:** An extensive literature search was performed to gather pertinent information on the topic of wound healing in patients with cancer. The effects that surgical procedures, radiation therapy, chemotherapy, and nutritional deficits play in wound healing in these patients were reviewed and collated. **Results:** The current knowledge and treatment of these aspects of wound healing in cancer patients are discussed, and observations and recommendations for optimal wound healing results are considered. **Conclusion:** Although wound healing may proceed in a relatively unimpeded manner for many patients with cancer, there is a potential for wound failure due to the nature and effects of the oncologic disease process and its treatments.

The physiologic changes that patients with cancer undergo as a part of the disease process itself, or in the course of treatment and recovery, are substantial and impact all aspects of their lives. With current treatment strategies aimed at multimodal protocols including surgery, radiation, and chemotherapeutic regimens, it is not surprising that wound healing becomes a matter of critical importance, regardless of whether the wound was surgically created, a complication of radio- or chemotherapy, or due to disease progression. Many patients undergo some form of surgical procedure during the course of their disease for diagnostic or staging purposes, treatment, or to manage complications of other treatment regimens.^1^ While the majority of wound healing is host-mediated, surgeons have the responsibility to provide wounds with a biologically favorable environment to facilitate wound healing and consequently should be aware of the many factors that interact in the healing of wounds, especially in patients with cancer.^2^

Wound healing is an orderly progression of carefully regulated, complex, interrelated processes that result in anatomic and functional integrity of tissues. Any processes, treatments, or interventions that modify or disrupt this progression can interfere with wound healing. Treating patients with cancer involves multiple modalities, all of which impact their ability to heal wounds. Surgery is often employed for curative purposes either by eradicating the tumor or by debulking tumor in anticipation of further treatment. Consequently, the timing of surgical intervention in relation to radio- or chemotherapy as well as the patient's nutritional status is fundamentally important in recovery from procedures and wound healing in general. Radiotherapy has a significant role in local control of disease or in the treatment of radiosensitive tumors; however, because it nonspecifically damages adjacent tissue in addition to cancer cells, it can further complicate wound healing. Chemotherapeutic regimens are frequently used as monotherapy or in conjunction with surgery and radiation in the treatment of cancer. These pharmacologic agents target proliferating cells, and can be used at any point in the treatment program—preoperatively, during the surgical procedure itself, or postoperatively as well. The ultimate goal of this multimodal approach to treating cancer is to eradicate disease while causing the least amount of harm to the patient; unfortunately, injury to the patient does occur, and the degree of damage to surrounding tissues impacts overall wound healing.

Consequently, it is crucial that physicians coordinate their treatment regimens to help optimize wound healing by understanding the effects of radiation, chemotherapy, and nutrition on wound healing in patients with cancer.

## RADIOTHERAPY

Radiation as a treatment modality is based on the ability of target tissues to absorb energy, which on the atomic scale causes excitation or “ionization” of electrons to higher energy orbits around the nucleus. Energy is released when these electrons return to their previous, more stable orbits, resulting in cellular damage to vital structures including DNA. The amount of radiation delivered to tissues is quantified in radiation absorbed doses (rad) or gray (Gy), with one Gy equivalent to 100 rad. Although the total dose of ionizing radiation administered in clinical settings is individualized on the basis of patient size and purpose, the amount of radiation can range from 0.25 millirad for a chest radiograph, to treatment doses of 4500 rad in radiation oncology, to near-tolerance doses of 7500 rad in bone marrow ablation procedures.

The biologic effect of radiation on tissues has been recorded as early as the 1890s, with its application in the treatment of cancer being described shortly thereafter. It was observed in 1906 that ionizing radiation was cytotoxic to the germinal cells of the testis while the mature Sertoli cells were left relatively undamaged.^3^ This ultimately led to the conclusion that the effects of radiation were more focused on dividing cells, which then was applied in the 1920s as a viable treatment option for cancer.^4^,^5^ However, the detrimental effect of ionizing radiation on healthy tissue was also becoming apparent, with the classic description of localized erythema and slow to heal skin ulcerations being reported.^6^

Multiple factors are involved in determining the cumulative effects of radiation on tissue: the total dose and duration of exposure, the oxygenation status of the tissue, and the position of the cells within the cell cycle are important considerations. Radiation exposure is a dose-dependent effect, with improved survival of tissues with lower amounts of exposure.^7^ Coutard demonstrated in 1934 that smaller, more frequent doses of radiation were more toxic to cancer cells with less damage to surrounding tissues than one large, single dose.^5^,^8^ This fact forms the basis for current protocols in radiation oncology, in which the cumulative dose is fractionated and protracted over a period of time to minimize overall toxicity. Most therapeutic radiation is given in 20 to 30 fractions of 200 to 300 rads, limited to cumulative doses of 6000 rads or less, over a 5- to 6-week period. Tissue oxygenation and perfusion is also important: hypoxic tissue is more radioresistant than vascularized tissue.^9^ On a similar note, actively dividing tissues are more susceptible to damage than resting cells: cells in the G2-M phases of the cell cycle are the most sensitive to radiation damage, while cells in the S phase are the least sensitive.^10^,^11^ In tumor biology, this observation is clinically significant, as rapidly dividing cancer cells will be more susceptible to radiation-induced cell damage; however, healthy tissues with high turnover rates such as epithelium or mucosa will also be injured, which ultimately can lead to delayed healing.

External beam radiation is the most commonly used modality in radiotherapy for cancer. The radiation beam is by necessity delivered through the skin and underlying structures before reaching its target tissue. Unfortunately, this results in unwanted radiation damage to the surrounding tissues along with the target tissue, resulting in early skin changes followed by chronic, progressive damage occurring more than 6 months after therapy has been completed. Acute findings are typically reversible and correlate with the total dose and frequency of radiation treatments: findings include erythema, dry desquamation followed by wet desquamation, ultimately progressing to skin necrosis.^6^ The early response to radiation is an inflammatory reaction with erythema, swelling, warmth, and tenderness; at doses greater than 4000 rads, moist desquamation with bullae and ulceration is sometimes seen.^12^ These ulcers typically heal after the conclusion of the treatment. Delayed or chronic effects are seen at approximately 6 months after radiation therapy, and can include changes in skin pigmentation, increasing fibrosis, sebaceous gland dysfunction, loss of hair follicles, telangiectasia of capillary beds, skin necrosis, and even tumor genesis.^6^,^13^ These chronic changes are usually irreversible.

Radiation-induced skin changes are the most visible effects on irradiated tissues. One of the first changes seen is dryness within the irradiated area. Once the squamous epithelium of the skin is damaged, the tissue sloughs off in either a dry or moist desquamation. Cell sloughing occurs within weeks after exposure due to the continued maturation of squamous cells without further cell division^14^; the rate of sloughing is dependent on the turnover of these growth-arrested cells. Different species have been demonstrated to slough skin cells at different rates: in humans, the turnover time for the epidermis ranges between 59 and 72 days.^15^,^16^ This process ultimately results in a thin, translucent appearance to the skin, absent hair follicles and sebaceous glands, and irregular pigmentation due to damaged melanocytes. The hypoxic, thin, dry epidermis also has lower tensile strength, making it susceptible to microscopic and macroscopic tears, which can lead to infection or chronic ulceration. This process further delays wound healing in irradiated tissues.^6^,^14^

In addition to skin structure changes, the underlying connective tissue also displays histologic changes in response to ionizing radiation. Acute changes in connective tissue include inflammation and edema of collagen bundles^17^ with deposition of a fibrinous exudate below the epidermis. This is followed by gradual replacement of adipose tissue and extracellular matrix by dense fibrous tissue and atypical fibroblasts.^14^ These cells do not proliferate as well as healthy fibroblasts, and deposit an immature collagen subtype. Even at time periods of 15 to 20 years following radiation exposure, fibroblasts have been found to contain intracellular structures such as degenerating mitochondria, multiple vacuoles, and irregular rough endoplasmic reticulum that indicate permanent damage.^18^ Clinically, this is evidenced by decreased wound tensile strength in comparison with controls, which further delays wound healing following radiotherapy.^19^

Radiation exposure has a detrimental effect on vascular structures as well. After exposure to ionizing radiation, superficial blood vessels initially dilate, resulting in erythematous skin changes. The arteries and arterioles within the irradiated tissue undergo loss of elasticity and progressive vascular sclerosis, resulting in chronic tissue hypoxia.^20^ Microscopically, the lumens of these vessels occlude with fibrin thrombi, leading to tissue edema. This loss of vascular supply continues to progress in a near linear fashion for the remainder of the patient's life, leading to long-term, localized deficits in wound healing in these patients.^21^,^22^ While most authors suggest that progressive microvascular occlusion is the primary cause of poor wound healing in irradiated tissues, others have suggested that damage to the basal layer of the epidermis may play a major role in chronic radiation damage.^18^ Another possibility is that radiation may have direct effects on other components of the wound, such as collagen, with vascular damage as a secondary occurrence.^15^,^18^,^19^

The complications of wound healing are worsened by radiation exposure to skin, connective tissue, and underlying vasculature. Clinically, prior radiation exposure has been demonstrated to increase the incidence of flap failures, fistulas, wound necrosis, delayed or prolonged healing, infection, and exposure of vital structures.^23^–^26^ Of note, wound healing is most affected by ionizing radiation when surgery is performed 6 months or more after radiation therapy. During the intervening period, the irradiated tissue becomes hypoxic, and fibroblasts become dysfunctional, which can lead to increased wound healing complications. Consequently, the timing of surgery in reference to proposed radiotherapy can have a significant impact on the outcome of wounds.^26^–^28^

Preoperative radiotherapy prior to surgical resection or tumor debulking is based on several tenets of tumor biology. Proponents suggest that manipulation of the tumor during surgical exploration may promote metastatic disease by “spillage” of viable cancer cells into the operative site or vasculature, leading to recurrence or dissemination.^29^ Preoperative radiation therapy may decrease the viability of tumor cells, making them less likely to survive or proliferate on spillage.^5^,^30^ However, this has not been definitively demonstrated: a randomized study involving preoperative versus postoperative radiation therapy on patients with head and neck cancer did not yield clinically significant improvement in outcomes.^28^ Another indication for preoperative radiation may be to decrease the overall tumor volume prior to surgical exploration, thus potentially allowing for total excision of a previously unresectable lesion. One caveat of this theory is that the volume of tissue excised in the surgical specimen may actually be higher following preoperative radiotherapy, due to the extensive scarring and fibrosis seen in tissues following radiation treatment.^24^,^31^ Also, a preoperative reduction in tumor volume locally may not necessarily correlate with a higher cure rate postoperatively.^32^

The effects of radiation on wound healing are primarily contingent on the total amount of radiation exposure as well as the timing and overall duration of treatment. Preoperative radiotherapy typically occurs 3 to 6 weeks prior to surgery. When doses larger than 50 Gy are administered, or treatments are given less than 3 weeks before surgery, a significant increase in wound complications results.^14^,^26^,^29^,^33^,^34^ In these situations, even the transfer of healthy tissue from outside of the irradiated field results in delayed wound healing in comparison with flap healing when postoperative radiotherapy is utilized.^35^

Fewer wound complications have been demonstrated when postoperative radiation therapy is selected.^6^ Because acute radiation exposure delays wound healing due to effects on the skin, connective tissue, and vasculature, it is logical to conclude that the effects on healing wounds can be minimized if the majority of wound healing has occurred prior to radiation treatment. If, however, wound healing is incomplete or the wound is infected when radiation therapy is initiated, further complications may arise. Although 3 weeks postoperatively has been deemed sufficient time for wound healing to progress prior to starting radiotherapy, this may be delayed by up to several weeks to allow for infection to resolve or complete but delayed healing to occur.^14^,^36^ Treatments should be initiated within 6 to 8 weeks postoperatively, as the benefits of radiotherapy may decrease if treatments are delayed further.^37^,^38^ Interestingly, Isaacs et al demonstrated that radiation therapy could be initiated in open wounds if “appropriate precaution” is taken.^38^ This includes ensuring adequate nutritional support and starting radiotherapy to surrounding tissues, preferably before 6 weeks have elapsed postoperatively.^38^ This concept challenges the principle of allowing wounds to completely heal before initiating radiotherapy in order to prevent further delay in necessary treatments. If local recurrence rates are similar in cases of preoperative versus postoperative radiation therapy in overall cancer treatment, it is preferable to perform radiotherapy postoperatively to minimize wound healing complications in this patient

## CHEMOTHERAPY

Chemotherapy, acting alone or in conjunction with surgery and radiation, is a fundamental treatment of cancer. This treatment modality targets proliferating cells by interfering with specific components of the cell cycle. When used in combination with surgery, chemotherapeutic regimens may be indicated preoperatively to decrease the tumor burden prior to surgical resection, or postoperatively to help eradicate residual disease. Although chemotherapeutic agents preferentially target rapidly dividing cells, any tissue can be affected by these treatments: macrophages and fibroblasts involved in wound healing are just as susceptible to these effects as cancer cells. Consequently, the timing of surgical intervention as well as the specific agents prescribed should be considered in wound healing in patients with cancer. Several classes of chemotherapeutic agents exist in current treatment protocols, and each class has characteristic effects on cancer cells and on wound healing as well.

### Alkylating agents

Alkylating agents such as cyclophosphamide (Cytoxan), chlorambucil (Leukeran), thiotepa (Thioplex), mechlorethamine (Mustargen), and cisplatin (Platinol) inhibit the cell cycle by alkylating DNA nucleotides, leading to cross-linking, strand breakage, and miscoding into RNA. While their application in different chemotherapeutic regimens is diverse, as a class these agents typically produce side effects involving the dividing cells of bone marrow, hair follicles, and gastrointestinal tract; their effects on wound healing are variable. Cyclophosphamide, for example, inhibits wound healing by attenuating the initial vasodilation and subsequent neovascularization during the proliferative phase of wound repair.^39^–^41^ Several studies in animal models indicate decreased wound tensile strength at doses of 165 to 500 mg/kg.^42^,^43^ However, the study by Mann et al did not demonstrate decreased wound strength at doses less than 100 mg/kg.^44^ The most deleterious effects on wound tensile strength were seen when 200 mg/kg or higher doses were administered 1 or 2 days postoperatively.^45^ Human studies using standard therapeutic doses of 20 to 30 mg/kg did not result in increased wound complications, however.^46^,^47^ Although high-dose cyclophosphamide has detrimental effects on wound healing in several animal studies, similar results in humans at therapeutic doses do not demonstrate this effect; consequently, initiating cyclophosphamide therapy at therapeutic doses below 100 mg/kg should minimize drug adverse effects in delaying postsurgical wound healing.

Thiotepa, like cyclophosphamide, inhibits fibroblast function and thus potentially can delay wound healing. Decreases in wound tensile strength have been demonstrated with administration of thiotepa at doses of 4 to 8 mg/kg in a rat model^48^; however, in other trials, doses of 0.4 mg/kg, 0.6 mg/kg, and 0.8 mg/kg did not result in delayed wound healing.^49^–^51^ Similarly, in human studies, therapeutic doses up to 0.8 mg/kg do not seem to impact wound healing significantly.^52^,^53^ These studies also indicate that the timing of thiotepa administration within the first 3 days of the postoperative period is also not critical, and therapeutic doses less than 0.8 mg/kg do not result in increased wound complications.^1^

Mechlorethamine or nitrogen mustard, as it is frequently referred, also negatively impacts fibroblast function, resulting in delayed wound healing.^54^,^55^ In animal studies, mechlorethamine doses between 0.3 and 0.6 mg/kg decreases wound tensile strength.^1^ Administration of this agent at the time of wounding leads to histologic evidence of impaired healing, including delayed fibroplasia, delayed endothelial proliferation, and delayed production of extracellular fibers, while infusion on the third postoperative day demonstrated both a diminished amount of granulation tissue and collagen content.^54^,^55^ However, patients with breast or colorectal cancers treated with 0.4 mg/kg of mechlorethamine cumulatively did not demonstrate an increase in wound or anastamotic complications.^56^ Interestingly, treating bowel with intraperitoneal mechlorethamine immediately following resection in patients with colon cancer also did not influence the incidence of wound dehiscence.^57^ Although animal studies seem to demonstrate detrimental effects of mechlorethamine on wound healing in the immediate postoperative period, studies in humans have not corroborated these results.

As with many of the other alkylating agents, cisplatin has been demonstrated in several animal studies to significantly impact wound healing. Even after a single dose of 5 mg/kg in rats, wound tensile strength is reduced when assessed on postoperative days 4, 7, 14, and 28.^58^ In an investigation by Engelmann et al, preoperative cisplatin in rats decreased fibroblast proliferation, reduced connective tissue proliferation, and inhibited neovascularization.^59^ These findings were validated in subsequent studies, which also demonstrated inhibition of the early proliferative phase of wound healing by cisplatin. In studies involving intestinal anastamoses, cisplatin-treated rats have a significantly lower anastamotic bursting pressure on postoperative days 7–10, although this effect was not clinically significant by postoperative day 14.^60^,^61^ The negative effects of cisplatin on wound healing can be attenuated with concurrent treatment with sodium thiosulfate, which lowers platinum levels in tissue and has demonstrated a protective effect against cisplatin toxicity when the agent is administered intraperitoneally rather than intravenously.^62^ Although further testing in human studies is warranted, experimentally administered cisplatin appears to have detrimental effects on the early proliferative phase of wound healing, especially with intravenous therapy.

### Antimetabolites

Antimetabolites, as a class, function by inhibiting purine and pyrimidine synthesis, which effectively limits DNA replication as well as RNA transcription. Agents such as methotrexate, 5-fluorouracil, 6-mercaptopurine, and azathioprine (Imuran) are frequent components of chemotherapeutic regimens for a variety of tumor types. Side effect profiles are characterized by gastrointestinal disturbances, nephrotoxicity, and bone marrow suppression. Methotrexate, for example, demonstrates a dose-dependent transient decrease in wound tensile strength when administered preoperatively.^42^,^54^,^63^ This effect is most pronounced on postoperative days 3–7, but tensile strength is equivalent to controls by postoperative day 21.^42^ Studies in which a leucovorin (folinic acid) “rescue” was administered in conjunction with methotrexate did not demonstrate an increase in wound complications, although these studies involved small patient populations that limit the power of the results obtained.^64^,^65^ Although animal studies suggest a relationship between the dosing and timing of methotrexate administration on postoperative wound healing, a similar effect has yet to be validated by larger scale human studies. Leucovorin “rescue” in animal studies, however, does seem to mitigate some of these negative effects on wound tensile strength.^63^

5-fluorouracil also demonstrates a similar decrease in wound tensile strength in rat studies when high perioperative doses are administered. This effect is amplified in malnourished rats with 20% to 25% weight loss, which is a dire predictor for patients with cancer, who tend to be catabolic.^66^ Several animal studies have attempted to evaluate the effect of 5-fluorouracil on visceral anastamoses, but these have yielded variable results, depending on the timing of 5-fluorouracil administration.^1^,^53^,^67^–^69^ In human subjects administered 60 mg/kg of 5-fluorouracil intravenously, contradictory results were obtained from 2 different studies: 1 study^70^ demonstrated an increase in wound morbidity when treatment occurred on postoperative days 7 and 10; the other study did not yield clinically significant differences when 5-fluorouracil was administered, beginning on postoperative day 14.^71^ Although results from animal and human studies are somewhat equivocal, the potentially negative effect of 5-fluorouracil on wound healing can be minimized when administered at least 14 days postoperatively in adequately nourished subjects.

The effects of 6-mercaptopurine and azathioprine on wound healing are less clear than the other antimetabolites. In the case of 6-mercaptopurine, administration of the agent produces a dose-related suppression of connective tissue organization, a decreased number of foreign body giant cells, and a derangement in the normal formation of capillaries in comparison with controls. However, the quantity of macrophages, mononuclear cells, and fibroblasts were unchanged, as was the amount of hydroxyproline in the extracellular matrix.^72^ Unfortunately, as wound tensile strength was not addressed in this study, the clinical relevance and potential application of these results are inconclusive.^1^,^69^ Similarly, although azathioprine has been tested in several animal studies, its effects in humans have not been addressed. In rats, wound healing was transiently suppressed on postoperative day 8, although this effect was negligible by day 15.^73^ Other studies in which azathioprine was administered in doses of 20 to 30 mg/kg per day yielded conflicting results in terms of wound tensile strength.^74^,^75^ Further study is warranted before definitive conclusions can be obtained regarding the effects of these agents on wound healing.

### Plant alkaloids

Plant alkaloids such as vincristine (Oncovin) and vinblastine (Velban) affect cell replication by de-polymerizing microtubule assemblies, effectively halting cell division during metaphase. These agents typically are associated with a degree of neurotoxicity and hyperuricemia, but do not cause significant bone marrow suppression. Studies in mice administered 3 mg/kg of vincristine at the time of surgery resulted in a transient decrease in wound tensile strength on postoperative day 3, which resolves by postoperative days 7 and 21.^42^ Studies in patients with Wilms' tumor who were treated with 1.0 to 1.5 mg/m^2^ of intravenous vincristine preoperatively did not demonstrate an increase in postoperative wound complications.^76^ When vincristine was administered in combination with doxorubicin (Adriamycin) and cisplatin preoperatively in sarcoma patients who later underwent surgical resection, there was no effect on wound morbidity postoperatively.^77^ Although a transient decrease in wound healing may occur with vincristine, this does not seem to increase wound complications in clinical studies.

### Antitumor antibiotics

Antitumor antibiotics are another category of agents used in chemotherapeutic regimens in the treatment of many different forms of cancer. These medications inhibit DNA-dependent RNA synthesis by interfering with transcription. Agents such as bleomycin (Blenoxane), doxorubicin, actinomycin D, and mitomycin C typically cause gastrointestinal dysfunction as well as bone marrow toxicity, although each medication also has its own side effect profile as well. Bleomycin limits skin fibroblast production of collagen, which helps account for its ability to impede wound healing.^78^ This effect was noted when 30 mg/kg of bleomycin administered postoperatively demonstrated decreased wound tensile strength, most noticeably around postoperative day 7, although similar findings were not evident 3 or 21 days postoperatively.^42^ This appears to be a dose-dependent and timing-related relationship, as bleomycin's negative effects on wound healing also decreased from the time of administration of the agent.^79^ Minimal changes are elicited when intralesional bleomycin is employed, and there is little evidence for morbidity associated with intracavitary bleomycin as well.^80^,^81^ While animal studies suggest that bleomycin may impair wound healing when dosed preoperatively or at the time of surgery, these effects may not be clinically significant by 3 weeks postoperatively.

The effects of the antitumor antibiotic doxorubicin on wound healing have been studied in several animal models. When administered in a single 6 mg/kg perioperative dose on the day of surgery or 3 days postoperatively, doxorubicin demonstrated a decrease in wound tensile strength up to 21 days postoperatively in a rat model.^42^,^82^ Similar results were obtained by Mullen et al,^83^ although the 6 mg/kg dose of doxorubicin utilized in these studies was supratherapeutic; therapeutic doses of the agent did not have the same negative effects.^69^ Another study utilizing a rabbit model demonstrated a significant decrease in wound tensile strength 14 days postoperatively when doxorubicin was dosed either 7 or 4 days preoperatively.^84^ Preoperative administration of this medication has the most profound effect on postoperative wound healing, especially when dosed 7 days prior to surgery; postoperatively administered, doxorubicin, however, does not seem to negatively impact subsequent wound healing.^83^,^85^ Lawrence et al demonstrated a decrease in wound tensile strength only on postoperative days 20 and 30 when 8 mg/kg of doxorubicin was administered 7 days postoperatively in a rat model.^86^ Combining doxorubicin therapy with radiation increases the incidence of wound complications, while another study demonstrates a possible reversal of these effects with the simultaneous administration of growth hormone.^85^,^87^ When administered immediately postoperatively or after wound healing has already occurred, doxorubicin treatment did not result in an increase in wound-related complications; however, administration of the agent should be avoided up to 7 days perioperatively, on the basis of delayed wound healing seen in animal models.^88^

Actinomycin D also demonstrates a transient effect on wound healing when studied in animal models. Postoperative administration of intraperitoneal actinomycin D at 0.6 mg/kg resulted in a 38% decrease in wound tensile strength on postoperative day 3; however, this resolves by postoperative day 21.^42^ Similarly, in rabbits, wound tensile strength is decreased on postoperative day 7 following topical administration of actinomycin D to surgically created wounds inoculated with cancer cells.^89^ While wound healing seems only transiently impaired by actinomycin D, more evidence exists for the negative effects of mitomycin C on wounds. When administered intraperitoneally in rats at doses ranging from 0.23 mg/kg to 0.41 mg/kg, mitomycin C did not affect the wound tensile strength of intestinal anastamoses; however, 2 mg/kg administered intraperitoneally decreased both collagen content and wound tensile strength in rats.^90^–^92^ Intravesical administration of the agent in human studies following bladder resection did not demonstrate an increase in wound complications, although bladder irritation was reported by a minority of patients.^93^ Mitomycin C, like doxorubicin, can result in soft tissue necrosis and skin ulceration after extravasation injuries, and animal studies demonstrate that intraperitoneal administration can delay wound healing in a dose-dependent manner.^94^

### Corticosteroids

Corticosteroids are frequently prescribed for patients with cancer, not as chemotherapeutic agents but to alleviate pain secondary to tumor growth and the accompanying inflammatory process. Corticosteroid therapy is a well-known impediment to wound healing, both acutely and following long-term use of this class of medication in the perioperative period by decreasing the inflammatory response and subsequent fibroplasia.^95^–^100^ Preoperative steroid therapy resulted in a 7.9% incidence of wound dehiscence in Green's review, with 29% of wounds having some complication of wound healing; other studies have estimated the incidence of wound dehiscence to be between 0.35% and 3.5% with concurrent steroid therapy.^101^ Studies in which corticosteroid therapy was delayed until 3 to 5 days postoperatively did not demonstrate a negative effects on wound tensile strength, however.^99^,^102^ In addition, coadministration of vitamin A seems to reverse some of the detrimental effects of steroids on wound healing: topical or systemic vitamin A stimulates monocytic inflammation, leading to fibroplasia and subsequent angiogenesis.^95^,^103^,^104^ While preoperative corticosteroid therapy has broad implications on postoperative wound healing over a wide range of doses and duration of treatment, delaying therapy until after the inflammatory phase of wound healing has subsided may help minimize these negative effects.

Overall, chemotherapeutic agents have varied effects on tissues, including interfering with metabolic processes, DNA structure or replication pathways, and cell division. As these agents target any rapidly growing tissues in addition to cancer cells, the skin and epithelial cells are susceptible to their potentially detrimental effects on wound healing. Considerations such as the cumulative dose and timing of administration during the perioperative period, the duration of exposure to these agents, and potentially additive effects of combination therapy with multiple agents also impact their overall potency and clinical effects. Currently, human studies have not demonstrated significant increases in wound complication rates from these agents as they are currently administered, either alone or in combination therapy with radiation and surgery. Delaying the initiation of chemotherapeutic regimens until 7 to 10 days postoperatively seems to have minimal effects on wound healing in this patient population.

## NUTRITION

Although many factors are involved in assessing overall health status in patients with cancer, nutrition plays a significant role in influencing tumor biology, comorbid conditions, and responses to treatment. The relationship between optimal wound healing and positive nutritional balance is well documented.^105^ Clinicians must consider their patients' overall nutritional status along with any therapeutic interventions being considered, whether that includes surgery, radiation, chemotherapy, or combinations of these modalities, to minimize complications and delays in wound healing in this patient population.

### Nutritional status in patients with cancer

In most studies assessing weight loss as the primary indicator of nutritional status in patients with cancer, approximately 40% to 80% of patients are malnourished, whether this is due to tumor biology, surgical procedures, radiation or chemotherapy, or psychological factors.^106^,^107^ Malnourished patients have a greater susceptibility to infectious complications along with delayed wound healing, which, in combination with their diseased state, significantly increases postoperative morbidity and mortality.^108^,^109^ Malnutrition also increases the metabolic activity of organs such as the liver, which physiologically borrows substrate from skin and muscle to maintain vital functions.^108^ Maintaining adequate nutritional balance in cancer patients undergoing treatment is essential to minimize the risk of complications such as enteric fistula formation, incisional wound dehiscence, as well as carotid rupture after head and neck surgery.^105^

Physiologic stressors such as infection and injury initiate a series of metabolic reactions leading to a negative nitrogen balance and ultimately a decrease in lean body mass, especially if patients do not have proper nutritional support. Although the initial metabolic event is a protective response to injury, if this stage is prolonged for extended periods of time, it becomes harmful to patients. This stress response, mediated by the complex interaction of cytokines, glucocorticoids, catecholamines, insulin, and insulin-like growth factors, can ultimately lead to a catabolic state if adequate nutritional support is not available.^107^

Many factors impact nutritional balance in patients with cancer, which can lead to increased morbidity and mortality during the course of their disease. Malnutrition has detrimental effects on cellular and humoral immunity in addition to negatively influencing tissue function and repair. Malnourished cancer patients have less tolerance for therapeutic interventions than patients in better nutritional states. Also, the tumor burden places increased demands on patients due to stress and tissue injury, which can lead only to further catabolic responses and nutritional depletion. In this patient population, timely and adequate nutritional supplementation is necessary to minimize the harmful consequences of malnutrition.^107^

Patients need adequate supplies of exogenous substrates to meet energy and protein requirements in order to protect visceral organs and attenuate catabolism of tissues such as muscle.^107^ Daily energy expenditure is associated with several components including basal metabolic rate, thermic effects of exercise, and thermogenic effects from food ingestion. Stress and illness in critically ill patients can increase basal energy expenditure by up to 40%. Similarly, cancer can induce localized tumor effects from rapid growth and division, as well as systemic effects due to metastatic disease, both of which can further increase the metabolic demands on an already critically ill patient. This can lead to glucose intolerance, increased fat depletion, and increased protein catabolism in these patients, which becomes more problematic as the tumor burden rises.^107^

### Nutritional management in the critically ill patient

Following acute injury, the body undergoes 3 phases of recovery in terms of metabolic activity: the early shock or ebb phase, followed by the catabolic or flow phase, culminating in the anabolic phase.^110^ Patients in the initial poststress period have an altered chemical environment characterized by increased levels of glucocorticoids, glucagon, and catecholamines, along with a decreased level of insulin. This metabolic milieu favors gluconeogenesis, glycogenolysis, and fatty acid oxidation. Protein derived from muscle is broken down to supply amino acid substrate for gluconeogenesis.^111^ This initial poststress state is not favorable for optimal substrate utilization; in fact, nutritional support is often unsuccessful during this period, and may even prove detrimental to the patient.^112^ Consequently, the goal of this phase is stabilization of the patient's cardiopulmonary and hemodynamic status: once this is achieved and the patient moves into the catabolic phase of recovery, nutritional support is then indicated. Although the exact timing of initiating enteral or parenteral feeding has not been entirely determined, it should not be delayed significantly past attainment of hemodynamic stability.^107^

Reasonable goals for nutritional support in the critically ill patient include minimizing starvation effects, preventing specific substrate deficiencies, and supporting the acute inflammatory response. These issues must be addressed until the hypermetabolic state has resolved and healing begins. Fluid and electrolyte balance should be attained, and an adequate amount of calories should be provided to meet the increased energy demands of the patient. Fluids and electrolytes must be judiciously administered to maintain adequate urine output and normal serum electrolyte levels. Similarly, vitamins and essential dietary elements must be supplemented according to recommended allowances. Periodic monitoring of serum electrolytes, liver enzymes, and lipid levels can ensure appropriate serum levels and helps avoid unnecessary complications.^107^ Although complete cessation of protein catabolism and wasting of lean body mass cannot be accomplished, the potential nitrogen deficit must be minimized to preserve vital structural proteins from breakdown into substrates.^107^

To objectively assess nutritional balance, several laboratory and clinical measures are available to identify patients at risk of malnourishment. Laboratory assessment of serum albumin, prealbumin, retinol-binding protein, and transferrin levels; cutaneous anergy testing of immune responsiveness and total lymphocyte count; and physical determinants like triceps skin fold measurement and lean body mass may be utilized and followed clinically.^113^ If these indicators are abnormal prior to surgical intervention, a nutritional support protocol is strongly recommended preoperatively to facilitate postoperative wound healing and minimize wound complications.^114^ Ultimately, the goal of preoperative support is to allow adequate protein synthesis postoperatively.^115^

One difficulty with nutritional support is accurately estimating the energy demands of the patient to provide adequate substrate to minimize the breakdown of energy stores. A minimum requirement of 25 to 30 kJ/kg per day is initially recommended for critically ill patients.^116^,^117^ Carbohydrates tend to be the primary source of nonprotein calories during the hypermetabolic state and can comprise 60% to 70% of the daily nutritional requirements. In situations where carbohydrates are either deficient from the diet due to inadequate support or less available for utilization as in patients with diabetes, protein degradation increases due to shunting of amino acid carbon skeletons toward gluconeogenesis. Similarly, the pentose shunt required for synthesis of ribose moieties for basic nucleic acid structure is also impaired in the context of carbohydrate unavailability, leading to attenuated cell activation and division.^105^

Daily fat requirements account for the additional 25% to 30% of nonprotein calories in hypermetabolic patients.^107^ Fat provides not only essential dietary fatty acids but also an energy-rich substrate to help meet metabolic demands. Studies of sepsis in patients with cancer indicate that calories from fat seem to be a preferred energy source, even in the presence of ample glucose stores.^118^–^120^ Essential fatty acid deficiency occurring in malnutrition limits 18-carbon precursors for arachidonic acid synthesis, which would negatively impact the inflammatory cascade at wound sites and in infected tissues.^105^,^121^

Similarly, protein needs are dramatically increased in critically ill patients.^107^ Increased catabolism in these patients is usually unresponsive to protein or glucose infusion; however, infusion of amino acids induces protein synthesis and allows for restoration of nitrogen balance.^122^,^123^ Studies in wounded animals demonstrate that diets deficient in certain amino acids resulted in more pronounced weight loss after injury and delayed healing of wounds. Essential amino acids including arginine and sulfur-containing amino acids are believed to be required for optimal wound healing and collagen deposition.^124^–^126^ These amino acids should be provided in adequate amounts to restore the nitrogen balance and limit muscle breakdown. In general, protein requirements in septic or injured patients range from 1.2 to 2.0 g/kg per /day, depending on the extent of injury and degree of physiologic stress. Typically, the suggested nonprotein calorie to nitrogen ratio is approximately 150:1; in these patients, however, higher levels of stress require increased protein needs in the range of 80:1 to 100:1.^107^

### Enteral and parenteral nutritional support

Nutritional supplementation in surgical patients involves enteral or parenteral routes. The general rule of thumb for postoperative nutrition favors enteral access: “If the gut works, use it.”^107^ (p 751) Enteral feeding is easily administered, well-tolerated, helps maintain the barrier mechanism of the gastrointestinal tract, and promotes mucosal growth and development.^107^ In animal studies, enteral feeding also demonstrated an important role in supporting the body's mucosal immune defenses.^127^,^128^ Enteral nutrition is more efficient, less expensive, and is associated with fewer metabolic complications like hyperglycemia, cholestasis, and fatty infiltration of the liver, all of which occur more frequently when the parenteral route is utilized.^129^ Several studies in surgical patients also support enteral nutrition if the gastrointestinal tract is functional: fewer infectious complications resulted when high-risk surgical and trauma patients were fed enterally in comparison with parenteral nutrition.^107^,^130^,^131^

Although the enteral route is preferred for nutritional support, certain clinical situations require alternate access. In patients who cannot receive enteral support due to mechanical risks like aspiration or functional deficits such as postoperative ileus, the parenteral route is an available option. Although total parenteral nutrition (TPN) requires central venous access through either peripherally or centrally inserted catheters, the risks of line placement and infection are outweighed by the benefits of adequate nutritional support. Although randomized, controlled studies have shown little or no benefit of TPN in well-nourished or slightly malnourished cancer patients undergoing treatments such as surgery, radiation, or chemotherapy,^107^ those patients with severe malnutrition prior to therapeutic interventions demonstrated improved outcomes following major elective surgery, major trauma, and, in some cases, bone marrow transplantation, when TPN was initiated preoperatively.^132^,^133^

Although a precise protocol detailing optimal nutritional therapy in patients with cancer does not exist, many attempts have been made to systematically categorize patients by nutritional status and determine appropriate nutritional support. Not surprisingly, different subsets of patients respond variably to enteral and parenteral nutrition sources, and clinical studies have been inconclusive regarding routes of administration.^107^ However, clinical findings were discussed in a conference sponsored by the National Institutes of Health, the American Society for Parenteral and Enteral Nutrition, and the American Society for Clinical Nutrition. Klein et al published the results, in which 33 prospective, randomized, controlled trials including more than 2500 surgical patients were reviewed to evaluate perioperative nutritional support. Most patients in this sample population had cancer, the majority of which were gastrointestinal malignancies. The panel concluded the following: (1) TPN administration to malnourished patients—determined by weight loss, plasma proteins, or prognostic indices—with gastrointestinal cancer 7 to 10 days prior to surgery reduces postoperative complications by 10%; (2) routine use of early postoperative TPN in malnourished general surgical patients who did not receive TPN prior to surgery actually increases postoperative complications by approximately 10%; and (3) postoperative support is mandatory for patients unable to eat for extended periods of time after surgery in order to prevent the harmful effects of starvation.^134^ Although the length of time starvation can be tolerated without a subsequent increase in morbidity is unknown, the panel did conclude that wound healing and recovery from surgery may be hindered if TPN administration is delayed longer than 5 to 10 days postoperatively in patients unable to tolerate enteral feeding.^134^ Clearly, additional studies are required to further determine the clinical indications for parenteral nutrition in the perioperative period, as well as the role of enteral support in the preoperative period for patients at risk of developing postoperative complications.

Patients with cancer need to obtain nutritional balance to avoid risks associated with malnutrition and complications of their treatment regimens. Nutritional management of patients should be incorporated into the overall treatment program but should not interfere with potentially life-saving or life-extending protocols. Cancer patients requiring surgical intervention, radiation, and chemotherapy in the management of their diseases clearly can benefit from improved nutritional and metabolic status to minimize potentially correctable causes of morbidity and mortality.

### Nutritional components for wound healing

In addition to meeting adequate calorie and protein dietary needs in this patient population, supplementation of several important vitamins and minerals is also required. These water-soluble and fat-soluble vitamins function as cofactors in many processes involved in wound repair and healing.^105^ Vitamins C and A, along with several other essential components, contribute to optimize wound healing and recovery. Vitamin C is necessary for hydroxylation of proline and lysine residues in collagen polymerization and cross-linking.^105^ Hydroxyproline is essential for the helical stability of collagen and its subsequent secretion into the extracellular matrix, while hydroxylysine is required for collagen cross-linking and further polymerization of fibers.^2^ In the absence of vitamin C, collagen formation is poor, and the unhydroxylated protein is susceptible to proteolysis.^135^ This leads to weakness of the extracellular matrix and the clinical condition scurvy, manifested by abnormal wound healing.^2^

Wolfer et al described a 50% reduction in the tensile strength of healing wounds in patients suffering from vitamin C depletion.^136^ Similarly, Crandon et al determined that one third of patients with wound dehiscence postoperatively were deficient in vitamin C.^137^ Depletion of vitamin C, as measured by plasma ascorbic acid levels, interrupts normal wound healing due to low tissue levels of ascorbic acid and the resultant decrease in wound tensile strength; however, normal wound healing can still be achieved if vitamin C is adequately replaced in the immediate postoperative period.^138^

Vitamin C also contributes to the body's resistance to infection.^2^ In studies performed by Meyer and Meyer, guinea pigs received subcutaneous injections of *Staphylococcus aureus*, and abscess formation was compared between vitamin C–deficient animals and nondeficient animals. In the vitamin C–deficient group, the abscesses formed were much larger than those in the control group, which was attributed to impaired collagen production in the test animals, leading to inadequate sequestration or “walling off” of the bacteria.^139^ Neutrophils are also affected in vitamin C deficiency: as vitamin C is required to reduce oxygen to superoxide, free radical production is decreased and neutrophil function is impaired.^2^

Although the role of vitamin C in wound healing is well recognized, there is no convincing evidence that supplementing vitamin C when tissue levels are normal further accelerates wound repair and healing. However, it is critical to administer vitamin C in critically ill or injured patients, as deficiency can rapidly develop because of minimal vitamin C stores in the body. Patients at risk of developing vitamin C deficiency should receive 1 to 2 g of vitamin C daily to optimize wound healing.^140^

Vitamin A is another essential cofactor that is elemental in the wound repair process. Higher tissue concentrations of vitamin A increases recruitment of macrophages into wounds, leading to more effective phagocytic activity and cell-mediated immunity.^2^ Vitamin A has also been demonstrated to reverse some of the anti-inflammatory effects of corticosteroids, which are frequent components of chemotherapeutic regimens. This may be attributed to the ability of vitamin A to reverse some of the stabilizing effects of corticosteroids on lysosomal membranes, thereby restoring an active component of the inflammatory response.^141^,^142^ Doses of 25,000 IU/oz applied topically to wounds daily have been shown to have some local benefit without compromising the systemic effects of corticosteroid therapy.^143^ Vitamin A deficiency leads to decreased collagen synthesis and cross-linking, decreased epithelialization, and slower rate of wound closure.^144^ Patients with severe injuries, malabsorption, or malnutrition are at risk of vitamin A deficiency and should receive dietary supplement of 25,000 IU/day of vitamin A.^2^

Unlike roles of vitamins C and A, the role vitamin E plays in wound healing is not as well defined. Structurally, vitamin E is similar to corticosteroids, and functions similarly to stabilize cell membranes. Although this may implicate vitamin E as being counterproductive to wound repair, several studies suggest that vitamin E is beneficial to wound healing.^145^ For example, rats fed on a vitamin E–deficient diet have impaired collagen synthesis, which implies that vitamin E may be an important cofactor in collagen cross-linking and polymerization.^146^ In another investigation, vitamin E was found to have inhibitory effects on hyaluronidase activity in humans, which suggests a beneficial role in extracellular matrix deposition.^147^ Although the contributions of vitamin E are not clearly defined, it is likely that it contributes toward the wound healing process.

Zinc, an essential cofactor for many enzymes including DNA and RNA polymerase, serves a variety of biologic functions important for wound healing, including a role in the biosynthetic pathway of vitamin A.^148^–^150^ Zinc deficiency leads to decreased DNA and RNA synthesis, impaired protein production, and decreased cell proliferation: in zinc-deficient animals, wound granulation tissue contains lower concentrations of collagen and noncollagenous proteins.^2^ Restoration of zinc levels to normal has been shown to return wound healing back to baseline.^2^ As with vitamin C, zinc supplementation has no therapeutic value unless a true zinc deficiency exists; in fact, zinc may be detrimental when present in excessive quantities, as it competitively competes with copper, a cofactor for lysyl oxidase.^151^,^152^ This can lead to decreased cross-linking in immature collagen.^2^ Consequently, it is important to recognize patients at risk of developing zinc deficiency and administer only moderate amounts of zinc. Patients with malnutrition, infection, severe injuries, malabsorption, or on long-term TPN should be supplemented with 220 mg of zinc every 8 hours.^2^

Lawrence et al described an interesting wound healing model in rats inoculated with 10^6^ cells of methylcholanthrene-induced sarcoma either 14 or 7 days prior to wounding of the skin or deeper structures. The tumor grows locally at the site of administration, generally does not metastasize, and produces increasingly severe metabolic derangements beginning approximately 2 weeks after tumor implantation, progressing to death in 5 weeks after inoculation. Although this study demonstrated an expected impairment in the healing of incisional wounds, the deeper wounds seemed to heal normally, in spite of a significant tumor burden that may alter other metabolic processes. The wound tensile strength began to decrease approximately 19 days after tumor cells were injected, which also corresponded to the appearance of other manifestations of cancer cachexia as well: decreased food intake and carcass weight loss, increased gluconeogenesis, and increased whole-body protein turnover have all been described. It appears that tumor-induced metabolic changes contribute to the deficiency in wound healing that seems to develop concurrently, implicating a cumulative effect of tumor biology and host response in impaired wound healing in this model.^1583^

Malnutrition is frequently encountered in patients with cancer and must be addressed clinically, as protein and calorie deficiencies impact all aspects of their treatment and recovery. This subset of patients respond poorly to surgery, with impaired healing of surgical wounds and anastamoses, leading to the increased incidence of wound and anastamotic complications.^154^–^156^ Consequently, patients must be given adequate nutritional support in the perioperative period to not only prevent weight loss and correct nitrogen balance but also support or improve immune function, facilitate tissue repair, and optimize wound healing.^2^,^157^

## CONCLUSION

Many factors impede the ability of patients with cancer to heal: the disease process itself, the timing and invasiveness of the treatments aimed at palliation or cure, and host factors including mental outlook, nutritional status, and social situation all play important roles in the management and outcome of their disease. The multimodal approach toward treating these patients includes a combination of surgical intervention, radiation therapy, and chemotherapeutic regimens, all of which can interact to delay wound healing in this population. Consequently, the timing of surgery in relation to radio- and chemotherapy, as well as the patient's overall nutritional status, is critical in the recovery from procedures and wound healing.

Radiotherapy plays a significant role in the local control of disease. However, because it nonspecifically damages adjacent tissues in addition to cancer cells, it can further complicate wound healing. Factors such as the total dose and duration of exposure, the oxygenation status of the tissue, and the position of cells within the cell cycle influence the cumulative effects of radiation. Because of radiation-induced injury to the skin, connective tissue, and underlying vasculature, wound healing is further complicated. Although preoperative radiation has its indications for reducing tumor burden or making an initially unresectable, radiosensitive lesion more amenable to surgical excision, the effects on the surrounding tissue can increase the incidence of wound complications if doses larger than 50 Gy are administered or if treatments are given less than 3 weeks prior to surgery. As fewer wound complications have been demonstrated when postoperative radiotherapy is selected, this treatment modality is preferred. Treatment can be delayed up to 6 to 8 weeks postoperatively to allow the majority of wound healing to occur before initiating radiation therapy.

Chemotherapeutic regimens target proliferating cells by interfering with specific components of the cell cycle. Although rapidly dividing tumor cells are the primary target, any tissues or cell types with high turnover rates—macrophages and fibroblasts involved in wound healing, for example—are susceptible to the effects of systemic or locally administered agents. Consequently, the timing of surgical intervention as well as the specific agents prescribed should be considered when managing wounds in patients with cancer. The effects of alkylating agents such as cyclophosphamide, thiotepa, mechlorethamine, and cisplatin have been demonstrated to decrease wound tensile strength and impede wound healing during the early proliferative phase in animal studies. However, most human studies at therapeutic doses of these agents have not yielded conclusive results. Similarly, antimetabolites such as methotrexate, 5-fluorouracil, 6-mercaptopurine, and azathioprine have negative effects on wound healing when administered during the perioperative period; delaying treatment with these agents until 2 weeks postoperatively may mitigate some of these effects. Plant alkaloids such as vincristine transiently decrease wound tensile strength in animal studies, but this does not appear to be a clinically significant impediment to wound healing in humans. The antitumor antibiotics, on the other hand, including agents such as bleomycin, doxorubicin, actinomycin D, and mitomycin C, have variable effects on wound healing, especially if treatment is initiated perioperatively. Studies on bleomycin and doxorubicin demonstrate negative effects on wound tensile strength and collagen synthesis that can be limited by avoiding treatment within 1 week of surgical intervention. Finally, the anti-inflammatory effects of corticosteroids can be minimized if treatment is held several days before and after surgery. The concurrent administration of vitamin A reduces some of these detrimental effects by stimulating monocyte infiltration, fibroplasia, and angiogenesis.

As an estimated 40% to 80% of patients with cancer are clinically malnourished, these patients are increasingly susceptible to infectious complications and delayed wound healing. This is further exacerbated by tumor biology and therapeutic interventions. These patients require adequate supplies of exogenous substrates to meet energy and protein requirements and attenuate the catabolism of vital tissues. A minimum requirement of 25 to 30 kJ/kg per day is recommended for critically ill patients, with carbohydrates comprising 60% to 70% of nonprotein calories and the remainder supplied by dietary fats, including 18-carbon precursors necessary for arachidonic acid synthesis. Protein requirements between 1.2 and 2.0 g/kg per day are also required, and should include essential amino acids such as arginine and methionine, both of which are necessary for optimal wound healing. The choice between enteral and parenteral nutrition is usually made on the basis of clinical indications; however, when possible, enteral supplementation is preferred, as studies have demonstrated fewer metabolic and infectious complications with enteral nutrition. Finally, deficiencies in antioxidants such as vitamins C and A, vitamin E, and zinc, all of which function as cofactors in the wound healing process, should be corrected with supplementation, as clinically indicated.
